# The landscape of Medicare policies for gender-affirming surgeries in Canada: an environmental scan

**DOI:** 10.1186/s12913-024-11361-w

**Published:** 2024-08-10

**Authors:** Dave Gwun, Brennan Snow, Emery Potter, Rachel Loewen Walker, Alexandra L. Millman, Yonah Krakowsky, Gianni R. Lorello, Janice Du Mont, Lucy C. Barker, Percy Lezard, Marudan Sivagurunathan, David R. Urbach, Kathleen Armstrong

**Affiliations:** 1 Temerty Faculty of Medicine, University of Toronto, Toronto, ON Canada; 2 Women’s College Research Institute, Women’s College Hospital, 76 Grenville Street, Toronto, ON M5S 1B2 Canada; 3 Lawrence S. Bloomberg Faculty of Nursing, University of Toronto, Toronto, ON Canada; 4 Department of Women’s and Gender Studies and Political Studies, University of Saskatchewan, Saskatoon, SK Canada; 5 Division of Urology, Department of Surgery, University of Toronto, Toronto, ON Canada; 6 Transition Related Surgery Program, Women’s College Hospital, Toronto, ON Canada; 7 Department of Anesthesiology and Pain Medicine, University of Toronto, Toronto, ON Canada; 8 Department of Anesthesiology and Pain Management, University Health Network - Toronto Western Hospital, Toronto, ON Canada; 9 The Wilson Centre, Toronto General Hospital, Toronto, ON Canada; 10 Dalla Lana School of Public Health, University of Toronto, Toronto, ON Canada; 11 Department of Psychiatry, University of Toronto, Toronto, ON Canada; 12 Department of Indigenous Studies, Law and Social Justice, Wilfrid Laurier University, Waterloo, ON Canada; 13 Department of Surgery, Women’s College Hospital, Toronto, ON Canada; 14 Division of Plastic and Reconstructive Surgery, Department of Surgery, University of Toronto, Toronto, ON Canada

**Keywords:** Insurance policy, Access to care, 2S-LGBTQ + health, Resource

## Abstract

**Background:**

Many studies have described barriers to gender-affirming surgery (GAS) in Canada; however, few have explored why these barriers persist. To address this knowledge gap, we sought to describe documents related to public health insurance (Medicare) for GAS to identify the types of procedures covered, variations in coverage across provinces and territories, and changes in policy over time.

**Methods:**

We conducted a descriptive cross-sectional study using an environmental scan approach. We queried 23 government websites, the Google search engine, and an online legal database between July 2022 and April 2024 to gather gray literature documents related to GAS and Medicare. Variables from relevant documents were compiled to create a present, at-glance overview of GAS Medicare coverage for all provinces and territories and a timeline of policy changes across Canada.

**Results:**

Eight provinces and three territories had documents or websites related to GAS Medicare coverage (85%). We identified 15 GAS procedures that were covered variably across Canada. Yukon (*n* = 14) covered the most types of GAS, while Quebec and Saskatchewan covered the least (*n* = 6). Mastectomy and genital surgeries were covered across Canada, but other GAS were rarely covered. Five provinces and territories provided coverage for travel-related costs. Our GAS Medicare timeline showed differential expansion of GAS coverage in Canada over the last 25 years.

**Conclusions:**

We provide previously unreported information regarding GAS Medicare coverage in Canada. We hope our findings will help patients and healthcare providers navigate a complicated public healthcare system. We also highlight barriers within GAS Medicare documents and make recommendations to alleviate those barriers.

**Supplementary Information:**

The online version contains supplementary material available at 10.1186/s12913-024-11361-w.

## Background

Transgender, nonbinary, or gender-diverse (TGD) is an inclusive term that encompasses Two-Spirit, transgender, nonbinary, and genderqueer, among other gender identities. In Canada, approximately one in 300 individuals aged 15 and older identify as transgender or nonbinary [1]. TGD individuals are at greater risk of poor mental health outcomes and negative healthcare experiences [2, 3]. Additionally, many TGD individuals experience gender dysphoria due to the incongruence between one’s gender identity and sex assigned at birth [4]. Gender-affirming care seeks to support TGD individuals through a comprehensive approach comprised of social, psychological, medical, and surgical interventions.

Gender-affirming surgery (GAS) is one component of gender-affirming care. It includes a range of feminizing and masculinizing procedures that align an individual’s anatomy with their gender identity. Chest (top) and genital (bottom) surgeries are among the most common GAS [5, 6]. Other GAS include facial surgeries, which alter an individual’s facial features, and voice surgery, which alters the length of the vocal cord to adjust its pitch. GAS is associated with improved health outcomes, including increased quality of life and reduced psychological distress [7–9]. Conversely, lack of access and delays in the provision of these services are associated with significant health risks and worsened mental health outcomes [2, 7].

While the number of GAS performed in Canada increases each year, barriers persist in terms of access and coverage [10, 11]. Notable barriers include information inaccessibility, financial and insurance concerns, and travel-related costs [6, 12–14]. While these barriers have been identified, we do not know why they persist in Canada’s public healthcare system. In Canada, public health insurance (Medicare) is regulated and administered by 13 provincial and territorial (jurisdictional) authorities who decide which services and procedures are covered within their jurisdiction. While the provincial and territorial Medicare plans share certain criteria and standards outlined in the Canadian Health Act, one’s medical coverage, including access to GAS, is ultimately dependent on the province or territory where they are a primary resident [15]. These Medicare documents and the complexities of the healthcare system can be challenging for TGD individuals and healthcare providers to navigate, especially when there is no centralized resource to find accurate and up-to-date information. To address this gap, we conducted an environmental scan to gather and describe documents related to GAS and Medicare in Canada. In doing so, we hope to synthesize a current, at-glance overview of GAS Medicare coverage for all provinces and territories. We also seek to generate evidence-informed recommendations to facilitate more equitable GAS access.

## Methods

### Study design

We conducted a descriptive cross-sectional study using an environmental scan approach to gather publicly available gray literature (e.g., government, institutional, legal, scholarly, newspaper, etc.) documents regarding GAS Medicare coverage in Canada [16, 17]. An environmental scan is defined as the process of gathering information to use for informed decision-making and strategic planning. Previous studies have performed environmental scans to elucidate health programs that impact specific communities and have informed decision-making and strategic planning in the areas of mental health and women’s health [18, 19].

This study received approval from the institutional research ethics board at Women’s College Hospital (REB 2023–0019-E). We reported this study using the Standards for Reporting Qualitative Research checklist (Appendix A) [20].

### The search of the gray literature

For a complete description of the definitions, search concepts and terms, and the search strategy, please see the *Supplementary Methods* (Appendix B). Guided by previous publications, an environmental scan was conducted through a series of internet searches across 23 government websites, the Google search engine (Google.com), and a Canadian legal online database (Canlii.org). The scope of this environmental scan was delineated by the concepts: “gender-affirming surgery” and “policy documents.” Two authors (B.S., D.G.) conducted the search from July 2022 through April 2024.

After the search, two authors (B.S. and D.G.) applied inclusion criteria to narrow the search results. The inclusion criteria were as follows: 1) written in English or French, 2) relevant to GAS and Medicare, and 3) applicable to Canada. Search results that did not meet the inclusion criteria were excluded. Any discrepancies between the authors were resolved through discussion until a consensus was reached.

### Data extraction

The following information was extracted from the documents included in our study: 1) document type (government, legal or court, scholarly, nongovernment (e.g., community health organization) or other), 2) jurisdictions (i.e. provinces and territories), 3) publication date, 4) GAS mentioned, 5) Medicare funding status for GAS mentioned (covered, covered under specific criteria, not covered), and 6) coverage for travel-related costs.

### Reporting provincial and territorial Medicare funding for GAS procedures

Data extracted from government documents and websites were compiled to create a list of GAS covered by Medicare for each province and territory in Canada. Three jurisdictions did not have publicly available GAS Medicare documents or websites, and one did not specify coverage for individual procedures. For these jurisdictions, we submitted an Access to Information and Privacy (ATIP) request or contacted a representative from the regional government to inquire about GAS coverage.

GAS covered in Canada were grouped into categories (masculinizing, feminizing, other) as described in Table 1. We also included two nonsurgical procedures in the GAS list. Hair removal was included because it is a preoperative procedure required for penile inversion vaginoplasty and phalloplasty. Fertility treatments were also included because they may allow TGD individuals who wish to have genetically related children to pursue bottom surgeries. Nonsurgical GAS procedures and those that did not fit into masculinizing or feminizing categories were grouped as “other.”

**Table 1:** Gender-affirming surgeries (GAS) and nonsurgical procedures covered by Medicare in Canada

Masculinizing		Feminizing		Other
Top	Bottom	Top	Bottom	
Mastectomy	Hysterectomy ± salpingo-oophorectomy	Breast augmentation	Orchiectomy ± scrotectomy	Facial surgery
Chest contouring	Metoidioplasty*		Vaginoplasty†	Tracheal surgery
Chest/breast reduction	Phalloplasty*			Body contouring
				Hair removal
				Gamete harvesting and preservation

The asterisk (*) notes masculinizing bottom surgeries that may include a combination of clitoral release, urethroplasty, scrotoplasty, glansplasty, and testicular and erectile implants. The dagger (†) indicates feminizing bottom surgery, which may include a combination of penectomy, clitoroplasty, labiaplasty, and vulvoplasty. Disclaimer: While some documents may list the above procedures as individual GAS (e.g., penectomy), they may only be approved for coverage when performed as a part of GAS bottom surgeries (e.g., penile deconstruction for penile inversion) and never as an isolated procedure

A GAS was considered “covered” if documents explicitly stated coverage (e.g., “phalloplasty is covered”) or if all the necessary procedures for that GAS were covered (e.g., “we cover urethroplasty, scrotoplasty, vaginectomy, and insertion of testicular and approved penile implants”). If a procedure was covered under specific eligibility criteria, it was classified as “covered under specific criteria.” A procedure was deemed “not covered” if documents explicitly excluded it from coverage. If a GAS was not mentioned in government documents or websites, its Medicare funding status was determined using data from other sources captured in our initial search. If the Medicare funding status remained unclear, we noted it as “unknown.” When possible, the Medicare funding status for each GAS was cross-referenced to other sources included in our search to ensure accuracy.

Jurisdictional Medicare funding for the GAS was reported using descriptive statistics (counts, medians, and percentages). The findings were also compiled to create a matrix indicating the procedures covered in each province and territory.

### Timeline of GAS Medicare coverage

Metadata (date of publication) and data extracted (date of change, changes in coverage and eligibility criteria) from government documents, news releases, newspaper or magazine articles, and court or legal documents were compiled to create a timeline of GAS Medicare funding in Canada.

## Results

### Search results

**Fig. 1:**
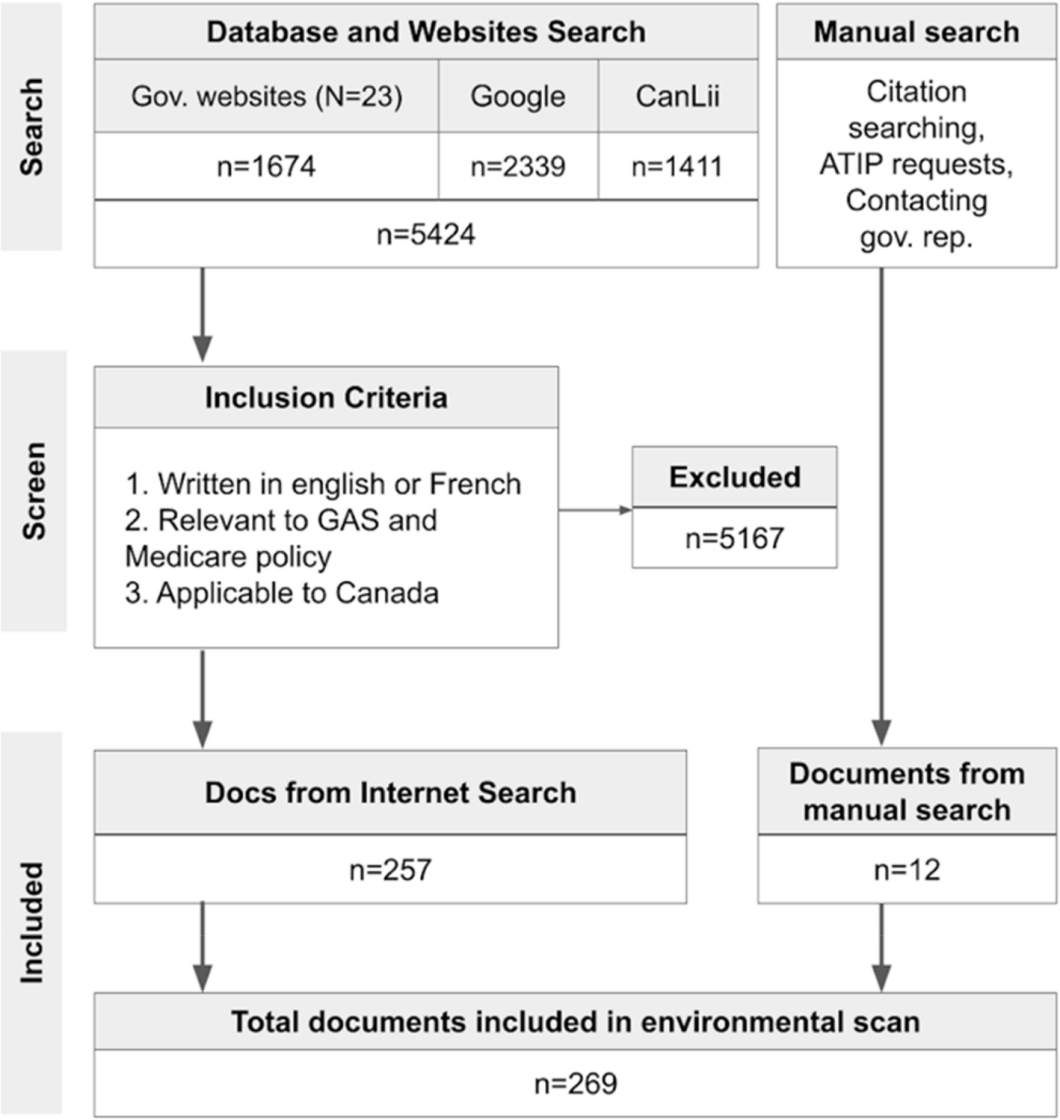
Flow chart of the environmental scan and screening results. This flow chart depicts the combined raw search results from 23 government (gov.) websites, the Google search engine (google.com), an online legal database (canlii.org), and the manual search. The online search produced 5424 results. After applying the inclusion criteria, 257 results were obtained. Twelve additional documents and webpages were gathered from the manual search, which consisted of citation searching, access to Information and Privacy (ATIP) requests, and contacting representatives from the regional government. In total, 269 documents were included in the environmental scan

The search produced 5428 results. After applying the inclusion criteria, 257 remained. Of these, 80 were government documents or websites, 28 were legal or court documents, 102 were newspaper or magazine articles, 16 were scholarly documents, 22 were nongovernment institutional resources, and nine were other. An additional 12 documents were identified through citation searching, ATIP requests, and contacting representatives from regional governmental health departments. In total, 269 documents and web pages were included in the environmental scan. (Fig. 1).

### Provincial and territorial GAS Medicare documents and websites

We found government documents or websites related to GAS Medicare coverage for 11 jurisdictions (85%). Manitoba, Nunavut, and Quebec did not have readily accessible GAS Medicare documents or websites. Nunavut’s GAS Medicare coverage was determined from a document retrieved from a representative of the Nunavut Health Department [21]. We could not retrieve documents for Manitoba and Quebec. Saskatchewan’s website stated GAS coverage but did not specify coverage for individual procedures [22]. Information regarding Medicare coverage for individual GAS procedures for Quebec, Manitoba, and Saskatchewan was gathered from a combination of news releases, newspaper articles, and community organization resources.

**Fig. 2:**
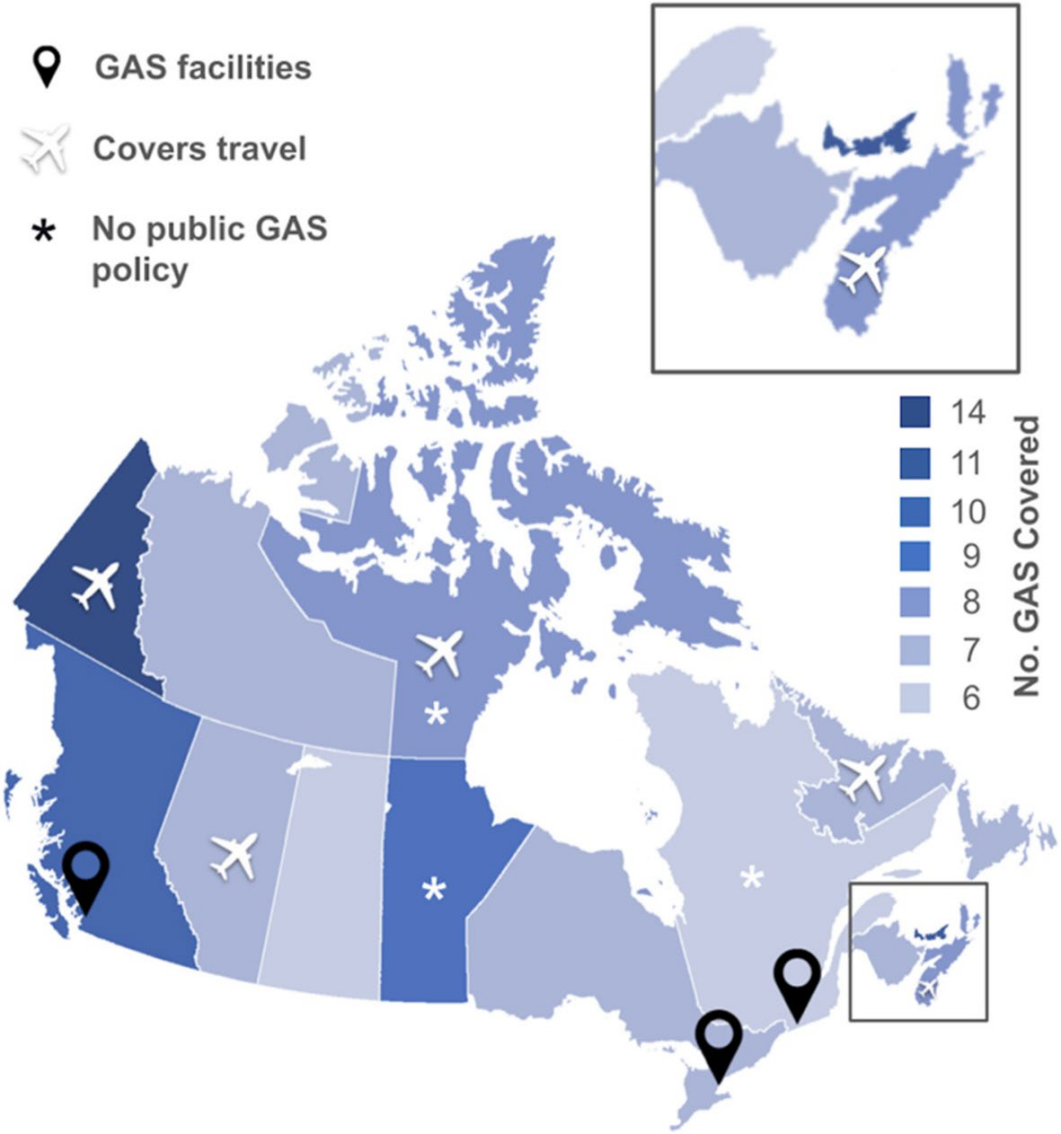
Geographic heat map showing the number of GAS covered by each province and territory. Map pins specify the locations where GAS are performed in Canada. From left to right: The Gender Surgery Program in Vancouver, British Columbia; Women’s College Hospital in Toronto, Ontario; and Centre Metropolitain de Chirurgie in Montreal, Quebec. The asterisk (*) specifies provinces without publicly available GAS Medicare policy documents or web pages. Provinces offering travel-related costs coverage (Alberta, British Columbia, Newfoundland and Labrador, Nova Scotia, and Nunavut) are indicated with an airplane symbol

**Fig. 3:**
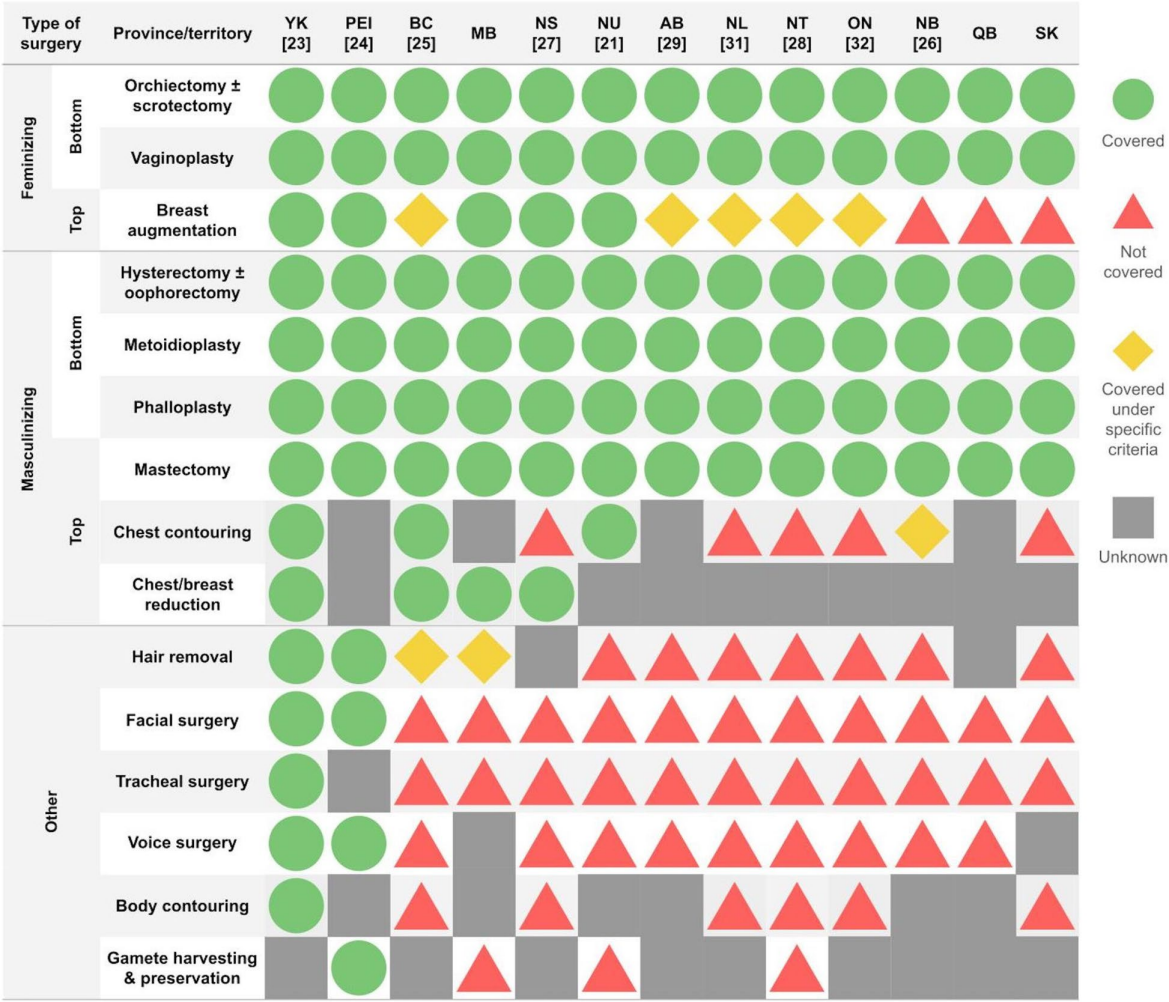
Summary of Medicare coverage for gender-affirming surgeries (GAS) for each province and territory in Canada. Provinces and territories are organized according to the most to least types of GAS covered. References to policy documents where the information is sourced are written in square brackets. The green circles indicate procedures covered by Medicare. Pink triangles identify procedures excluded from Medicare. Yellow diamonds indicate procedures that are covered under specific criteria. The gray squares denote unknown coverage. Abbreviations: AB = Alberta; BC = British Columbia; MB = Manitoba; NB = New Brunswick; NL = Newfoundland and Labrador; NS = Nova Scotia; NU = Nunavut; NWT = Northwest Territories; ON = Ontario; PEI = Prince Edward Island; QB = Quebec; SK = Saskatchewan; Yk = Yukon

We found 13 different GAS and two nonsurgical gender-affirming procedures that were covered variably across Canada. (Table 1) All 13 jurisdictions in Canada provided coverage for GAS to varying degrees. (Figs. 2 and 3) The median number of GAS covered was seven (range = 6–14). Yukon [23] provided coverage for the most number of GAS (*n* = 14), followed by Prince Edward Island [24] (PEI, *n* = 11) and British Columbia [25] (BC, *n* = 10). Saskatchewan and Quebec covered the fewest GAS (*n* = 6).

### Provincial and territorial coverage of masculinizing GAS

Masculinizing GAS covered in Canada included mastectomy, chest contouring, chest or breast reduction, and hysterectomy with or without salpingo-oophorectomy, metoidioplasty, and phalloplasty. (Fig. 3) All jurisdictions in Canada provided coverage for mastectomy and masculinizing bottom surgeries. Chest contouring was covered by BC [25], Nunavut [21], and Yukon [23]. New Brunswick [26], (NB) covered chest contouring only when performed with mastectomies. Gender-affirming chest/breast reduction was covered in BC [25], Manitoba, Nova Scotia [27], (NS) and Yukon [23].

### Provincial and territorial coverage of feminizing GAS

Feminizing GAS covered in Canada included breast augmentation, orchiectomy with or without scrotectomy, and vaginoplasty. All provinces and territories covered orchiectomy with or without scrotectomy and vaginoplasty. (Fig. 3) Breast augmentation was the next most covered feminizing procedure, funded in 10 jurisdictions. However, four jurisdictions only covered it if there was minimal or disproportionate breast growth after 12 months of continuous hormone therapy, and the Northwest Territories [28] only covered it if there was no breast growth after 18 months of hormone therapy.

### Provincial and territorial coverage of other GAS

Other GAS covered in Canada included facial surgery, voice surgery, tracheal surgery, body contouring, hair removal, and fertility treatments (e.g., gamete harvesting and preservation). (Fig. 3) Facial and vocal surgeries were covered by PEI [24] and Yukon [23]. Yukon [23] was the only jurisdiction to cover body contouring and tracheal surgery. PEI [24] and Yukon [23] covered hair removal. Manitoba only covered electrolysis hair removal only when performed by an approved provider and BC only covered hair removal for the portion of the graft as a prerequisite for urethral lengthening [25]. PEI was the only province to include and cover gamete harvesting and preservation as a gender-affirming procedure [24].

### Provincial and territorial coverage of travel-related costs

Five jurisdictions provided full or partial coverage of travel expenses related to GAS. (Fig. 2) Alberta [29], NS [27], and Yukon [30] provided coverage for out-of-province travel. Newfoundland and Labrador [31] and Nunavut [21] referred to provincial travel programs to which patients could apply. BC [25], Ontario [32], NB [26], and PEI [24], Saskatchewan [22] explicitly excluded travel-related costs from Medicare.

### Timeline of GAS Medicare coverage updates in Canada

**Fig. 4:**
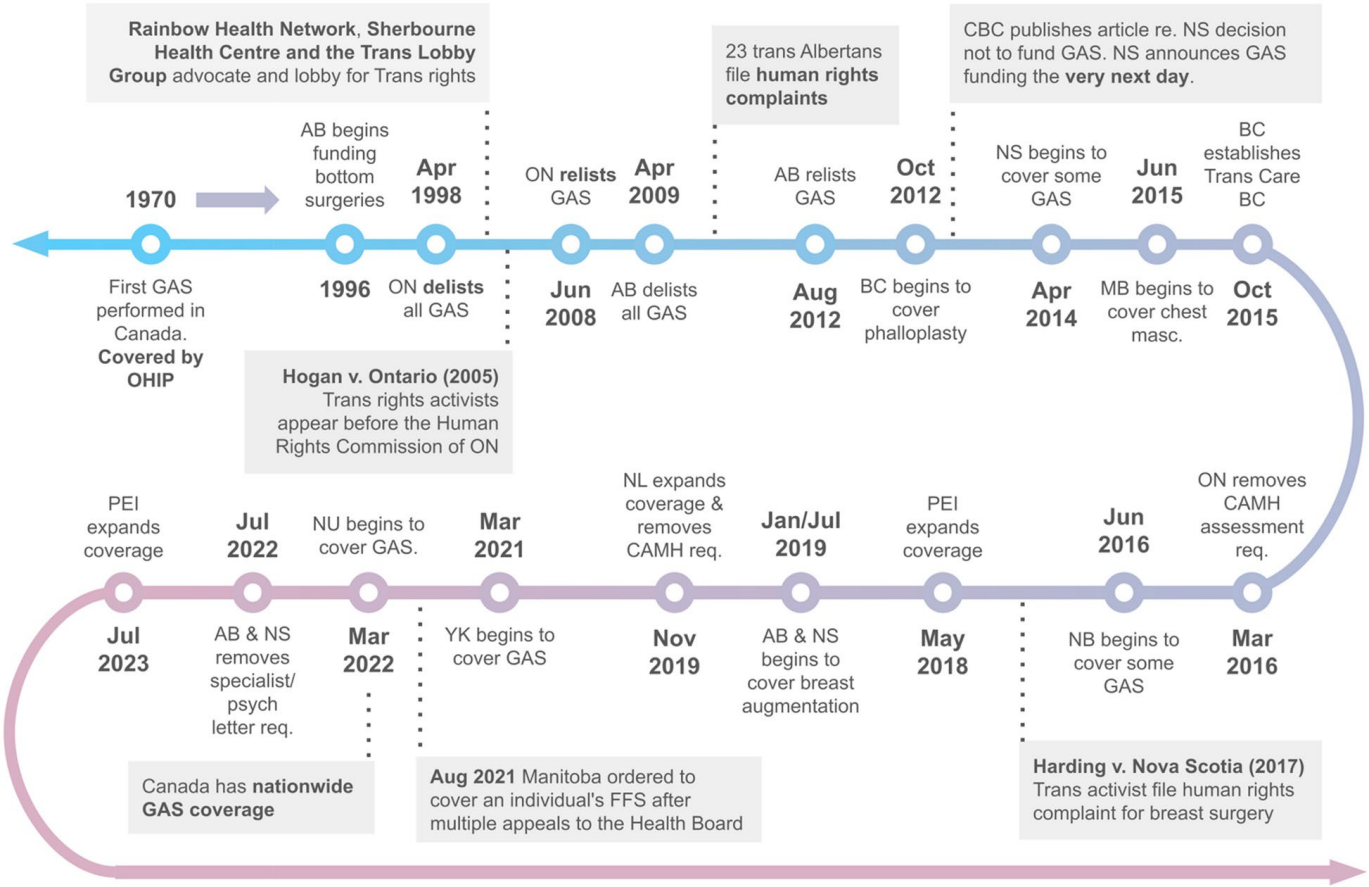
Timeline of gender-affirming surgery Medicare policy changes from 1970 to 2024. Abbreviations: AB = Alberta; Apr = April; Aug = August; BC = British Columbia; CAMH = Centre of Addiction and Mental Health; CBC = Canadian Broadcasting Channel; Feb = February; FFS = facial feminization surgery; GAS = gender-affirming surgery; Jan = January; Jul = July; Jun = June; Mar = March; masc. = masculinization; MB = Manitoba; NB = New Brunswick; NL = Newfoundland and Labrador; Nov = November; NS = Nova Scotia; NU = Nunavut; Oct = October; OHIP = Ontario Health Insurance Plan; ON = Ontario; PEI = Prince Edward Island; psych = psychologist; re. = regarding; req. = requirement; v. = versus; YK = Yukon

Figure 4 summarizes changes to GAS Medicare coverage. Ontario was the first province in Canada to perform and cover GAS in 1970 [33]. However, in 1998, Ontario delisted GAS from coverage and did not relist it until 2008 [34]. Alberta also delisted GAS from coverage in 2009 and relisted it in 2012 [35, 36]. NS, PEI, and NB began covering GAS in 2014, 2015, and 2016, respectively [37–39]. In 2021, Yukon expanded its coverage to align with the World Professional Association for Transgender Health Standard of Care 7 (WPATH SOC 7) guidelines [40, 41]. The same year, Manitoba covered an individual’s facial feminization surgery for the first time [42]. In 2022, Nunavut was the last jurisdiction in Canada to provide GAS coverage [43]. In 2023, PEI expanded their GAS coverage to include facial and chest feminization surgeries [44].

## Discussion

Here, we conducted an environmental scan to provide a comprehensive overview of Medicare for GAS in Canada. 11 jurisdictions had documents or websites stating Medicare coverage for GAS. In total, our search identified 13 GAS and two nonsurgical procedures as covered variably across Canada. While all provinces and territories provided coverage for mastectomy and bottom surgeries, the remaining GAS were rarely covered. Five jurisdictions provided coverage for travel-related costs necessary for GAS. Our timeline of GAS Medicare documents showed that coverage for GAS has changed considerably in the last 25 years. Overall, our findings highlight the complex and dynamic nature of GAS Medicare documents in Canada.

Our results demonstrate the need to improve patient navigation and enhance information accessibility for TGD individuals seeking GAS. Four jurisdictions did not have readily accessible documents regarding GAS Medicare coverage of individual procedures. The absence of these documents acts as a barrier for TGD individuals seeking GAS by restricting access to accurate and up-to-date information [45, 46]. For many TGD individuals, information seeking is the first step in determining what GAS they want and how to access them [12]. Unfortunately, TGD individuals cannot always rely on their primary care provider for information, and many report feeling confused about how to navigate the healthcare system and where to access information [12, 45]. Thus, online resources are essential for informing TGD individuals about the procedures available and covered in their home province or territory [47]. In light of this, we recommend that jurisdictions create readily available documents and websites regarding GAS Medicare if one does not already exist. We also recommend employing health system navigators whom TGD individuals contact for information to improve their experience in the healthcare system [48] (Table 2).

**Table 2:** Strategies to improve patient navigation and information accessibility for transgender, nonbinary, and gender-diverse (TGD) individuals seeking gender-affirming surgeries (GAS)

1. Create readily available documents and websites regarding provincial and territorial GAS Medicare policies
2. Employ health system navigators whom TGD individuals can contact to seek information

Our findings also highlight the need to expand and unify existing GAS Medicare coverage across Canada. While all jurisdictions covered mastectomy and bottom surgeries, access to other GAS was restricted due to limitations on the types of GAS covered. Notably, facial surgeries were only covered by two provinces, even though facial features play an essential role in the safety, quality of life, and mental health of TGD individuals [49–51]. In the United States, financial and insurance issues were the most common barriers to GAS [52–54]. Our results suggest similar barriers exist in Canada despite the country’s publicly funded healthcare system due to prohibitive GAS Medicare policies [47, 55]. Additionally, inconsistent GAS Medicare documents can act as barriers in themselves by complicating pathways to care and reimbursement [12]. These differences can lead to differential access to GAS, delays in care, unexpected costs, and postoperative dissatisfaction [6, 12–14, 56]. While introducing a uniform federal GAS Medicare document or website may alleviate these disparities, the nature of Canada’s public healthcare system makes it challenging to introduce health policies at the federal level because the delivery of health and other social services is the responsibility of the provincial and territorial governments. In lieu of a unifying federal GAS policy, we recommend that each province and territory expand their GAS Medicare documents to align with the most recent WPATH SOC and unify documents across Canada to ensure comprehensive and universal GAS access. (Table 3).

**Table 3:** Recommendations for gender-affirming surgeries (GAS) Medicare policies to improve access to GAS across Canada

1. Expand GAS Medicare policies to align with professional guidelines and standards such as the World Professional Association for Transgender Health Standards of Care
2. Unify GAS Medicare policies to reduce barriers to care and ensure equitable access to GAS across Canada

Finally, we would like to acknowledge the past activism and advocacy done by TGD communities in this area. While the exact reasons remain unknown, we believe that GAS coverage has advanced considerably in the last 25 years, largely because of the relentless efforts of TGD individuals, advocacy groups, and care providers who fought for expanded GAS Medicare coverage. For example, the Rainbow Health Network and the Trans Health Lobby group played a considerable role in relisting GAS in 2008 [57] Similarly, TGD activists in NS garnered media attention to pressure the government regarding the lack of GAS coverage, which quickly led to its funding [58–60]. Although the provincial and territorial governments ultimately have the responsibility to cover medically necessary surgery, our environmental scan suggests that community advocacy, political pressure, and court and human rights challenges based on discrimination are among the most effective ways of implementing change. Unfortunately, this places an unfair burden on TGD communities to advocate for adequate health care. Thus, we recommend that policymakers proactively address gaps in GAS Medicare policies and consult with the local TGD community and experts within the gender-affirming space performing active patient care when considering Medicare coverage changes. Also, these changes must be effectively communicated to TGD individuals and healthcare providers to avoid misinformation regarding GAS Medicare. (Table 4).

**Table 4:** Recommendations for policymakers working on gender-affirming surgeries (GAS) Medicare policies

1. Proactively address gaps in GAS Medicare policy documents to alleviate the burden on TGD communities
2. Consult with the local transgender, nonbinary, and gender-diverse (TGD) communities and experts within the gender-affirming space performing active patient care when considering Medicare coverage changes
3. Ensure that all GAS Medicare policy changes are effectively communicated to TGD individuals and healthcare providers to avoid misinformation

### Study limitations and future directions

This study has potential limitations. First, we used an environmental scan approach, which relied on Google and website searches to uncover Medicare-related documents for GAS in Canada. This approach may not capture documents that are not publicly available or have a poor web presence. It is also sensitive to search engine algorithms, which may vary results based on search date, IP address, and search history. Second, GAS Medicare documents and websites sometimes use ambiguous terminology when referencing GAS procedures. This may contribute to the underreporting of some GAS procedures. However, to avoid misinformation for patients and healthcare providers, we added disclaimers or reported the coverage as unknown when the procedure or its Medicare funding status was ambiguous. Third, while the authors believe summarizing policies and legislating regarding GAS is the first step towards equitable access to care, this work does not speak to the nuances associated with the process of seeking GAS, such as the administrative burden when applying for coverage, the surgeries that require out-of-province or territory travel, and the wait times associated with each surgery. Finally, government documents are subject to frequent updates and revisions, and GAS Medicare policies are bound to change after the publication of our article. In light of this, future works will focus on acquiring funding from governmental and institutional organizations to translate these findings into a readily accessible online resource that our team members can regularly update to ensure that TGD individuals have the most up-to-date information regarding GAS Medicare coverage. Additionally, this resource must be disseminated in a multitude of ways, including but not limited to information handouts, personal websites, social media accounts, academic journals, and conference presentations to reach prime targets.

This study was part of a larger environmental scan. The next steps include conducting semistructured interviews with stakeholders, including policymakers, government consultants, care providers and activists involved in implementing, changing or advising on GAS Medicare policies. The findings and limitations presented here will considered while designing and conducting our forthcoming study, which will investigate questions related to GAS Medicare policies not addressed in the present study, such as the process of policy development, factors that contributed to the differential expansion of GAS Medicare coverage across different provinces and territories, and challenges with implementing GAS Medicare policies into procedure and practice.

## Conclusion

Our study provides previously unreported information regarding GAS Medicare coverage in Canada, detailing coverage by province and territory to help patients and healthcare providers navigate a complicated public healthcare system. We also highlight barriers within the current landscape of GAS Medicare documents and make recommendations to alleviate those barriers. We hope that policymakers and politicians will respond to our recommendations for improving information accessibility and navigation, as well as expanding and unifying Medicare coverage for GAS across Canada. In this regard, leadership from Yukon and PEI has set a precedent for other jurisdictions to improve their Medicare coverage and align with the WPATH SOC 8 standards. At a time when regions are rolling back rights to TGD communities, we need increased clarity and coordination in coverage and practices nationwide.

### Supplementary Information

Supplementary Material 1.

Supplementary Material 2.

## Data Availability

The data are available upon reasonable request.

## Abbreviations

TGD: Transgender, nonbinary, or gender-diverse
GAS: Gender-affirming surgery
AITP: Access to information and privacy
BC: British Columbia
NB: New Brunswick
NT: Northwest Territories
NS: Nova Scotia
PEI: Prince Edward Island
WPATH SOC 7: World Professional Association for Transgender Health Standard of Care 7
