# Supplementary material for: The landscape of Medicare policies for gender-affirming surgeries in Canada: an environmental scan
Source: BMC Health Serv Res. 2024 Aug 10;24:916. doi: 10.1186/s12913-024-11361-w (PMC11316376; doi:10.1186/s12913-024-11361-w)
Supplement: Supplementary file 2 — Supplementary Material 2. [file 12913_2024_11361_MOESM2_ESM.docx]

**Supplementary Methods**

1. *Definitions*

Guided by previous publications, the scope of the environmental scan was delineated by the concepts: “gender-affirming surgery” and “policy documents.” We define gender-affirming surgery as any surgery that seeks to align an individual’s anatomy with their gender identity, including but not limited to feminizing top surgery (i.e., breast augmentation), feminizing bottom surgeries (i.e., orchiectomy with or without scrotectomy, vaginoplasty, vulvoplasty), masculinizing top surgeries (mastectomy, chest contouring, chest reduction), and masculinizing bottom surgeries (hysterectomy, salpingo-oophorectomy, vaginectomy, metoidioplasty, phalloplasty, scrotoplasty). (1) Policy documents are defined as follows: 1) public health legislation (provincial and territorial-specific Medicare legislation), 2) goals and objectives of federal, provincial, and territorial health ministries, 3) policies, standards, and guidelines specific to GAS for provinces and territories (e.g., insurance, billing schedule, fee). (2,3)

1. *Search concepts, terms, and synonyms*

Search concepts were used to create a list of search terms and synonyms to conduct the environmental scan. A list of concepts, terms and synonyms are listed in Supplementary Table 1.

| Supplementary Table 1: list of concepts and terms and synonyms for Environmental scan | | | |
| --- | --- | --- | --- |
| Concepts | “Gender-affirming surgery” | “Policy documents" | “Canada, provinces, territories” |
| Terms & synonyms | gender confirmation surgery | Insurance | Canada |
|  | gender-confirming surgery | Fee | Ontario |
|  | gender-affirming surgery | Billing | Manitoba |
|  | gender reassignment surgery |  | Saskatchewan |
|  | transition-related surgery |  | Alberta |
|  | sex reassignment surgery |  | British Columbia |
|  | gender change surgery |  | Yukon |
|  | sex change surgery |  | Northwest Territories |
|  | Transgender surgery |  | Nunavut |
|  | Transgender surgery |  | Quebec |
|  |  |  | Newfoundland and Labrador |
|  |  |  | New Brunswick |
|  |  |  | Nova Scotia |
|  |  |  | Prince Edward Island |

1. *Search of the grey literature*

The environmental scan was conducted through a series of internet searches to gather publicly available grey literature documents (e.g. government documents and webpages, legal and court documents, scholarly articles, and newspaper and magazine articles) [(1,2)](https://www.zotero.org/google-docs/?1h3EKa). The strategy was developed through an iterative process. New search terms were added to the search strategy (i.e. synonyms) as they came up. The search strategy was revised until the search produced key documents that were expected based on previous knowledge in this area. [(3)](https://www.zotero.org/google-docs/?9GqdnR) The search was conducted across multiple websites, search engines and online databases by two authors (B.S. & D.G.) between July 2022 and April 2024 (Figure 1):

(1) an online query of each of the provincial and territorial government or health department websites to retrieve policies surrounding coverage for GAS;

(2) an online query of CanLii (https://www.canlii.org/en/) to search for legal documents, such as human rights complaints, court cases, and commentary regarding GAS;

(3) an online scan using the Google search engine (https://www.google.com/) to supplement the above search for absent sources and collect additional sources not included in the search, such as news articles and community organization resources;

(4) an informal citation search that reviewed reference lists and associated links of documents and webpages found through the above methods and document requests.

1. *Search strategy & results*

The tables below list the websites, search engines, and online databases queried, the search strategy used, and the search results.

| Government, Ministry of Health, and Health Department websites | | | |
| --- | --- | --- | --- |
| Province | Site | Search terms | Results |
| Ontario | [Ontario.ca](http://ontario.ca) | ("gender confirmation" \| "gender confirming" \| "gender-affirming" \| "gender affirming" \| "gender reassignment" \| "transition-related" \| "sex reassignment" \| "gender change" \| "sex change" \| "transgender") AND ("surgery" \| "surgeries") | 84 |
| Ontario | [Ontario.ca](http://ontario.ca) | ("gender confirmation" \| "gender confirming" \| "gender-affirming" \| "gender affirming" \| "gender reassignment" \| "transition-related" \| "sex reassignment" \| "gender change" \| "sex change" \| "transgender") AND ("surgery" \| "surgeries") AND ("Insurance" \| "Fee" I "Billing") | 53 |
| Ontario | [ontariohealth.ca](http://ontariohealth.ca) | ("gender confirmation" \| "gender confirming" \| "gender-affirming" \| "gender affirming" \| "gender reassignment" \| "transition-related" \| "sex reassignment" \| "gender change" \| "sex change" \| "transgender") AND ("surgery" \| "surgeries") | 2 |
| Ontario | [ontariohealth.ca](http://ontariohealth.ca) | ("gender confirmation" \| "gender confirming" \| "gender-affirming" \| "gender affirming" \| "gender reassignment" \| "transition-related" \| "sex reassignment" \| "gender change" \| "sex change" \| "transgender") AND ("surgery" \| "surgeries") AND ("Insurance" \| "Fee" I "Billing") | 0 |
| Manitoba | [manitoba.ca](http://manitoba.ca) | ("gender confirmation" \| "gender confirming" \| "gender-affirming" \| "gender affirming" \| "gender reassignment" \| "transition-related" \| "sex reassignment" \| "gender change" \| "sex change" \| "transgender") AND ("surgery" \| "surgeries") | 94 |
| Manitoba | [manitoba.ca](http://manitoba.ca) | ("gender confirmation" \| "gender confirming" \| "gender-affirming" \| "gender affirming" \| "gender reassignment" \| "transition-related" \| "sex reassignment" \| "gender change" \| "sex change" \| "transgender") AND ("surgery" \| "surgeries") AND ("Insurance" \| "Fee" I "Billing") | 9 |
| Manitoba | [gov.mb.ca](http://gov.mb.ca) | ("gender confirmation" \| "gender confirming" \| "gender-affirming" \| "gender affirming" \| "gender reassignment" \| "transition-related" \| "sex reassignment" \| "gender change" \| "sex change" \| "transgender") AND ("surgery" \| "surgeries") | 175 |
| Manitoba | [gov.mb.ca](http://gov.mb.ca) | ("gender confirmation" \| "gender confirming" \| "gender-affirming" \| "gender affirming" \| "gender reassignment" \| "transition-related" \| "sex reassignment" \| "gender change" \| "sex change" \| "transgender") AND ("surgery" \| "surgeries") AND ("Insurance" \| "Fee" I "Billing") | 15 |
| Saskatchewan | [saskatchewan.ca](http://saskatchewan.ca) | ("gender confirmation" \| "gender confirming" \| "gender-affirming" \| "gender affirming" \| "gender reassignment" \| "transition-related" \| "sex reassignment" \| "gender change" \| "sex change" \| "transgender") AND ("surgery" \| "surgeries") | 12 |
| Saskatchewan | [saskatchewan.ca](http://saskatchewan.ca) | ("gender confirmation" \| "gender confirming" \| "gender-affirming" \| "gender affirming" \| "gender reassignment" \| "transition-related" \| "sex reassignment" \| "gender change" \| "sex change" \| "transgender") AND ("surgery" \| "surgeries") AND ("Insurance" \| "Fee" I "Billing") | 0 |
| Alberta | [alberta.ca](http://alberta.ca) | ("gender confirmation" \| "gender confirming" \| "gender-affirming" \| "gender affirming" \| "gender reassignment" \| "transition-related" \| "sex reassignment" \| "gender change" \| "sex change" \| "transgender") AND ("surgery" \| "surgeries") | 218 |
| Alberta | [alberta.ca](http://alberta.ca) | ("gender confirmation" \| "gender confirming" \| "gender-affirming" \| "gender affirming" \| "gender reassignment" \| "transition-related" \| "sex reassignment" \| "gender change" \| "sex change" \| "transgender") AND ("surgery" \| "surgeries") AND ("Insurance" \| "Fee" I "Billing") | 38 |
| Alberta | [albertahealthservices.ca](http://albertahealthservices.ca) | ("gender confirmation" \| "gender confirming" \| "gender-affirming" \| "gender affirming" \| "gender reassignment" \| "transition-related" \| "sex reassignment" \| "gender change" \| "sex change" \| "transgender") AND ("surgery" \| "surgeries") | 28 |
| Alberta | [albertahealthservices.ca](http://albertahealthservices.ca) | ("gender confirmation" \| "gender confirming" \| "gender-affirming" \| "gender affirming" \| "gender reassignment" \| "transition-related" \| "sex reassignment" \| "gender change" \| "sex change" \| "transgender") AND ("surgery" \| "surgeries") AND ("Insurance" \| "Fee" I "Billing") | 1 |
| British Columbia | [gov.bc.ca](http://gov.bc.ca) | ("gender confirmation" \| "gender confirming" \| "gender-affirming" \| "gender affirming" \| "gender reassignment" \| "transition-related" \| "sex reassignment" \| "gender change" \| "sex change" \| "transgender") AND ("surgery" \| "surgeries") | 169 |
| British Columbia | [gov.bc.ca](http://gov.bc.ca) | ("gender confirmation" \| "gender confirming" \| "gender-affirming" \| "gender affirming" \| "gender reassignment" \| "transition-related" \| "sex reassignment" \| "gender change" \| "sex change" \| "transgender") AND ("surgery" \| "surgeries") AND ("Insurance" \| "Fee" I "Billing") | 45 |
| British Columbia | [phsa.ca](http://www.phsa.ca/transcarebc/) | ("gender confirmation" \| "gender confirming" \| "gender-affirming" \| "gender affirming" \| "gender reassignment" \| "transition-related" \| "sex reassignment" \| "gender change" \| "sex change" \| "transgender") AND ("surgery" \| "surgeries") | 218 |
| British Columbia | [phsa.ca](http://www.phsa.ca/transcarebc/) | ("gender confirmation" \| "gender confirming" \| "gender-affirming" \| "gender affirming" \| "gender reassignment" \| "transition-related" \| "sex reassignment" \| "gender change" \| "sex change" \| "transgender") AND ("surgery" \| "surgeries") AND ("Insurance" \| "Fee" I "Billing") | 3 |
| Nunavut | [gov.nu.ca](http://gov.nu.ca) | ("gender confirmation" \| "gender confirming" \| "gender-affirming" \| "gender affirming" \| "gender reassignment" \| "transition-related" \| "sex reassignment" \| "gender change" \| "sex change" \| "transgender") AND ("surgery" \| "surgeries") | 1 |
| Nunavut | [gov.nu.ca](http://gov.nu.ca) | ("gender confirmation" \| "gender confirming" \| "gender-affirming" \| "gender affirming" \| "gender reassignment" \| "transition-related" \| "sex reassignment" \| "gender change" \| "sex change" \| "transgender") AND ("surgery" \| "surgeries") AND ("Insurance" \| "Fee" I "Billing") | 0 |
| Nunavut | [irespectmyself.ca](http://irespectmyself.ca/) | ("gender confirmation" \| "gender confirming" \| "gender-affirming" \| "gender affirming" \| "gender reassignment" \| "transition-related" \| "sex reassignment" \| "gender change" \| "sex change" \| "transgender") AND ("surgery" \| "surgeries") | 2 |
| Nunavut | [irespectmyself.ca](http://irespectmyself.ca) | ("gender confirmation" \| "gender confirming" \| "gender-affirming" \| "gender affirming" \| "gender reassignment" \| "transition-related" \| "sex reassignment" \| "gender change" \| "sex change" \| "transgender") AND ("surgery" \| "surgeries") AND ("Insurance" \| "Fee" I "Billing") | 0 |
| NWT | [gov.nt.ca](http://gov.nt.ca) | ("gender confirmation" \| "gender confirming" \| "gender-affirming" \| "gender affirming" \| "gender reassignment" \| "transition-related" \| "sex reassignment" \| "gender change" \| "sex change" \| "transgender") AND ("surgery" \| "surgeries") | 28 |
| NWT | [gov.nt.ca](http://gov.nt.ca) | ("gender confirmation" \| "gender confirming" \| "gender-affirming" \| "gender affirming" \| "gender reassignment" \| "transition-related" \| "sex reassignment" \| "gender change" \| "sex change" \| "transgender") AND ("surgery" \| "surgeries") AND ("Insurance" \| "Fee" I "Billing") | 3 |
| NWT | [nthssa.ca](https://www.nthssa.ca/) | ("gender confirmation" \| "gender confirming" \| "gender-affirming" \| "gender affirming" \| "gender reassignment" \| "transition-related" \| "sex reassignment" \| "gender change" \| "sex change" \| "transgender") AND ("surgery" \| "surgeries") | 0 |
| NWT | [nthssa.ca](https://www.nthssa.ca/) | ("gender confirmation" \| "gender confirming" \| "gender-affirming" \| "gender affirming" \| "gender reassignment" \| "transition-related" \| "sex reassignment" \| "gender change" \| "sex change" \| "transgender") AND ("surgery" \| "surgeries") AND ("Insurance" \| "Fee" I "Billing") | 0 |
| Yukon | [yukon.ca](http://yukon.ca) | ("gender confirmation" \| "gender confirming" \| "gender-affirming" \| "gender affirming" \| "gender reassignment" \| "transition-related" \| "sex reassignment" \| "gender change" \| "sex change" \| "transgender") AND ("surgery" \| "surgeries") | 90 |
| Yukon | [yukon.ca](http://yukon.ca) | ("gender confirmation" \| "gender confirming" \| "gender-affirming" \| "gender affirming" \| "gender reassignment" \| "transition-related" \| "sex reassignment" \| "gender change" \| "sex change" \| "transgender") AND ("surgery" \| "surgeries") AND ("Insurance" \| "Fee" I "Billing") | 2 |
| New Brunswick | [gnb.ca](http://gnb.ca) | ("gender confirmation" \| "gender confirming" \| "gender-affirming" \| "gender affirming" \| "gender reassignment" \| "transition-related" \| "sex reassignment" \| "gender change" \| "sex change" \| "transgender") AND ("surgery" \| "surgeries") | 63 |
| New Brunswick | [gnb.ca](http://gnb.ca) | ("gender confirmation" \| "gender confirming" \| "gender-affirming" \| "gender affirming" \| "gender reassignment" \| "transition-related" \| "sex reassignment" \| "gender change" \| "sex change" \| "transgender") AND ("surgery" \| "surgeries") AND ("Insurance" \| "Fee" I "Billing") | 3 |
| Nova scotia | [novascotia.ca](http://novascotia.ca/) | ("gender confirmation" \| "gender confirming" \| "gender-affirming" \| "gender affirming" \| "gender reassignment" \| "transition-related" \| "sex reassignment" \| "gender change" \| "sex change" \| "transgender") AND ("surgery" \| "surgeries") | 70 |
| Nova scotia | [novascotia.ca](http://novascotia.ca/) | ("gender confirmation" \| "gender confirming" \| "gender-affirming" \| "gender affirming" \| "gender reassignment" \| "transition-related" \| "sex reassignment" \| "gender change" \| "sex change" \| "transgender") AND ("surgery" \| "surgeries") AND ("Insurance" \| "Fee" I "Billing") | 6 |
| Nova scotia | [nshealth.ca](https://www.nshealth.ca/) | ("gender confirmation" \| "gender confirming" \| "gender-affirming" \| "gender affirming" \| "gender reassignment" \| "transition-related" \| "sex reassignment" \| "gender change" \| "sex change" \| "transgender") AND ("surgery" \| "surgeries") | 68 |
| Nova scotia | [nshealth.ca](https://www.nshealth.ca/) | ("gender confirmation" \| "gender confirming" \| "gender-affirming" \| "gender affirming" \| "gender reassignment" \| "transition-related" \| "sex reassignment" \| "gender change" \| "sex change" \| "transgender") AND ("surgery" \| "surgeries") AND ("Insurance" \| "Fee" I "Billing") | 4 |
| Prince Edward island | [princeedwardisland.ca](http://princeedwardisland.ca) | ("gender confirmation" \| "gender confirming" \| "gender-affirming" \| "gender affirming" \| "gender reassignment" \| "transition-related" \| "sex reassignment" \| "gender change" \| "sex change" \| "transgender") AND ("surgery" \| "surgeries") | 76 |
| Prince Edward island | [princeedwardisland.ca](http://princeedwardisland.ca) | ("gender confirmation" \| "gender confirming" \| "gender-affirming" \| "gender affirming" \| "gender reassignment" \| "transition-related" \| "sex reassignment" \| "gender change" \| "sex change" \| "transgender") AND ("surgery" \| "surgeries") AND ("Insurance" \| "Fee" I "Billing") | 4 |
| Newfoundland and Labrador | [www.newfoundlandlabrador.com](http://www.newfoundlandlabrador.com/) | ("gender confirmation" \| "gender confirming" \| "gender-affirming" \| "gender affirming" \| "gender reassignment" \| "transition-related" \| "sex reassignment" \| "gender change" \| "sex change" \| "transgender") AND ("surgery" \| "surgeries") | 0 |
| Newfoundland and Labrador | [www.newfoundlandlabrador.com](http://www.newfoundlandlabrador.com) | ("gender confirmation" \| "gender confirming" \| "gender-affirming" \| "gender affirming" \| "gender reassignment" \| "transition-related" \| "sex reassignment" \| "gender change" \| "sex change" \| "transgender") AND ("surgery" \| "surgeries") AND ("Insurance" \| "Fee" I "Billing") | 0 |
| Newfoundland and Labrador | [nlhealthservices.ca](http://nlhealthservices.ca) | ("gender confirmation" \| "gender confirming" \| "gender-affirming" \| "gender affirming" \| "gender reassignment" \| "transition-related" \| "sex reassignment" \| "gender change" \| "sex change" \| "transgender") AND ("surgery" \| "surgeries") | 0 |
| Newfoundland and Labrador | [nlhealthservices.ca](http://nlhealthservices.ca) | ("gender confirmation" \| "gender confirming" \| "gender-affirming" \| "gender affirming" \| "gender reassignment" \| "transition-related" \| "sex reassignment" \| "gender change" \| "sex change" \| "transgender") AND ("surgery" \| "surgeries") AND ("Insurance" \| "Fee" I "Billing") | 0 |
| Newfoundland and Labrador | [gov.nl.ca](http://gov.nl.ca) | ("gender confirmation" \| "gender confirming" \| "gender-affirming" \| "gender affirming" \| "gender reassignment" \| "transition-related" \| "sex reassignment" \| "gender change" \| "sex change" \| "transgender") AND ("surgery" \| "surgeries") | 55 |
| Newfoundland and Labrador | [gov.nl.ca](http://gov.nl.ca) | ("gender confirmation" \| "gender confirming" \| "gender-affirming" \| "gender affirming" \| "gender reassignment" \| "transition-related" \| "sex reassignment" \| "gender change" \| "sex change" \| "transgender") AND ("surgery" \| "surgeries") AND ("Insurance" \| "Fee" I "Billing") | 13 |
| Quebec | [quebec.ca](http://quebec.ca) | ("gender confirmation" \| "gender confirming" \| "gender-affirming" \| "gender affirming" \| "gender reassignment" \| "transition-related" \| "sex reassignment" \| "gender change" \| "sex change" \| "transgender") AND ("surgery" \| "surgeries") | 4 |
| Quebec | [quebec.ca](http://quebec.ca) | ("gender confirmation" \| "gender confirming" \| "gender-affirming" \| "gender affirming" \| "gender reassignment" \| "transition-related" \| "sex reassignment" \| "gender change" \| "sex change" \| "transgender") AND ("surgery" \| "surgeries") AND ("Insurance" \| "Fee" I "Billing") | 0 |
| Quebec | [gouv.qc.ca](http://gouv.qc.ca) | ("gender confirmation" \| "gender confirming" \| "gender-affirming" \| "gender affirming" \| "gender reassignment" \| "transition-related" \| "sex reassignment" \| "gender change" \| "sex change" \| "transgender") AND ("surgery" \| "surgeries") | 17 |
| Quebec | [gouv.qc.ca](http://gouv.qc.ca) | ("gender confirmation" \| "gender confirming" \| "gender-affirming" \| "gender affirming" \| "gender reassignment" \| "transition-related" \| "sex reassignment" \| "gender change" \| "sex change" \| "transgender") AND ("surgery" \| "surgeries") AND ("Insurance" \| "Fee" I "Billing") | 1 |

**CanLii**

| Database: https://www.canlii.org/ | | |
| --- | --- | --- |
| Province | Search terms | Results |
| Ontario | ("gender confirmation" OR "gender confirming" OR "gender-confirming" OR "gender-affirming" OR "gender affirming" OR "gender reassignment" OR "transition-related" OR "sex reassignment" OR "gender change" OR "sex change" OR "transgender") AND ("surgery" OR "surgeries") AND "Canada" AND ("Ontario" OR "O.N." OR "ON") | 323 |
| Manitoba | ("gender confirmation" OR "gender confirming" OR "gender-confirming" OR "gender-affirming" OR "gender affirming" OR "gender reassignment" OR "transition-related" OR "sex reassignment" OR "gender change" OR "sex change" OR "transgender") AND ("surgery" OR "surgeries") AND "Canada" AND ("Manitoba" OR "M.B." OR "MB") | 104 |
| Saskatchewan | ("gender confirmation" OR "gender confirming" OR "gender-confirming" OR "gender-affirming" OR "gender affirming" OR "gender reassignment" OR "transition-related" OR "sex reassignment" OR "gender change" OR "sex change" OR "transgender") AND ("surgery" OR "surgeries") AND "Canada" AND ("Saskatchewan" OR "S.K." OR "Sask.") | 92 |
| Alberta | ("gender confirmation" OR "gender confirming" OR "gender-confirming" OR "gender-affirming" OR "gender affirming" OR "gender reassignment" OR "transition-related" OR "sex reassignment" OR "gender change" OR "sex change" OR "transgender") AND ("surgery" OR "surgeries") AND "Canada" AND ("Alberta" OR "A.B." OR "AB") | 159 |
| British Columbia | ("gender confirmation" OR "gender confirming" OR "gender-confirming" OR "gender-affirming" OR "gender affirming" OR "gender reassignment" OR "transition-related" OR "sex reassignment" OR "gender change" OR "sex change" OR "transgender") AND ("surgery" OR "surgeries") AND "Canada" AND ("British Columbia" OR "B.C." OR "BC") | 200 |
| Nunavut | ("gender confirmation" OR "gender confirming" OR "gender-confirming" OR "gender-affirming" OR "gender affirming" OR "gender reassignment" OR "transition-related" OR "sex reassignment" OR "gender change" OR "sex change" OR "transgender") AND ("surgery" OR "surgeries") AND "Canada" AND ("Nunavut" OR "NT" OR "N.T.") | 31 |
| NWT | ("gender confirmation" OR "gender confirming" OR "gender-confirming" OR "gender-affirming" OR "gender affirming" OR "gender reassignment" OR "transition-related" OR "sex reassignment" OR "gender change" OR "sex change" OR "transgender") AND ("surgery" OR "surgeries") AND "Canada" AND ("Northwest Territories" OR "NWT" OR "N.W.T.") | 46 |
| Yukon | ("gender confirmation" OR "gender confirming" OR "gender-confirming" OR "gender-affirming" OR "gender affirming" OR "gender reassignment" OR "transition-related" OR "sex reassignment" OR "gender change" OR "sex change" OR "transgender") AND ("surgery" OR "surgeries") AND "Canada" AND ("Yukon" OR "YK") | 69 |
| New Brunswick | ("gender confirmation" OR "gender confirming" OR "gender-confirming" OR "gender-affirming" OR "gender affirming" OR "gender reassignment" OR "transition-related" OR "sex reassignment" OR "gender change" OR "sex change" OR "transgender") AND ("surgery" OR "surgeries") AND "Canada" AND ("New Brunswick" OR "N.B." OR "NB") | 69 |
| Nova Scotia | ("gender confirmation" OR "gender confirming" OR "gender-confirming" OR "gender-affirming" OR "gender affirming" OR "gender reassignment" OR "transition-related" OR "sex reassignment" OR "gender change" OR "sex change" OR "transgender") AND ("surgery" OR "surgeries") AND "Canada" AND ("Nova Scotia" OR "N.S." OR "NS") | 88 |
| Prince Edward island | ("gender confirmation" OR "gender confirming" OR "gender-confirming" OR "gender-affirming" OR "gender affirming" OR "gender reassignment" OR "transition-related" OR "sex reassignment" OR "gender change" OR "sex change" OR "transgender") AND ("surgery" OR "surgeries") AND "Canada" AND ("Prince Edward Island" OR "P.E.I." OR "PEI") | 45 |
| Newfoundland and Labrador | ("gender confirmation" OR "gender confirming" OR "gender-confirming" OR "gender-affirming" OR "gender affirming" OR "gender reassignment" OR "transition-related" OR "sex reassignment" OR "gender change" OR "sex change" OR "transgender") AND ("surgery" OR "surgeries") AND "Canada" AND ("Newfoundland and labrador" OR "N.L." OR "NL") | 41 |
| Quebec | ("gender confirmation" OR "gender confirming" OR "gender-confirming" OR "gender-affirming" OR "gender affirming" OR "gender reassignment" OR "transition-related" OR "sex reassignment" OR "gender change" OR "sex change" OR "transgender") AND ("surgery" OR "surgeries") AND "Canada" AND ("Quebec" OR "QB" OR "Q.B.") | 144 |

**Google search engine**

| Database: Google.ca | | |
| --- | --- | --- |
| Province | Search terms | Results |
| Prince Edward Island | ("gender confirmation" \| "gender confirming" \| "gender-confirming" \| "gender-affirming" \| "gender affirming" \| "gender reassignment" \| "transition-related" \| "sex reassignment" \| "gender change" \| "sex change" \| "transgender") AND ("surgery" \| "surgeries") AND "Canada" AND ("Prince Edward Island" \| "PEI" \| "P.E.I.") | 171 |
| Nova Scotia | ("gender confirmation" \| "gender confirming" \| "gender-confirming" \| "gender-affirming" \| "gender affirming" \| "gender reassignment" \| "transition-related" \| "sex reassignment" \| "gender change" \| "sex change" \| "transgender") AND ("surgery" \| "surgeries") AND "Canada" AND ("Nova Scotia" \| "NS" \| "N.S.") | 183 |
| New Brunswick | ("gender confirmation" \| "gender confirming" \| "gender-confirming" \| "gender-affirming" \| "gender affirming" \| "gender reassignment" \| "transition-related" \| "sex reassignment" \| "gender change" \| "sex change" \| "transgender") AND ("surgery" \| "surgeries") AND "Canada" AND ("New Brunswick" \| "NB" \| "N.B.") | 146 |
| Newfoundland and Labrador | ("gender confirmation" \| "gender confirming" \| "gender-confirming" \| "gender-affirming" \| "gender affirming" \| "gender reassignment" \| "transition-related" \| "sex reassignment" \| "gender change" \| "sex change" \| "transgender") AND ("surgery" \| "surgeries") AND "Canada" AND ("Newfoundland and Labrador" \| "NL" \| "N.L."\| ) | 148 |
| Quebec | ("gender confirmation" \| "gender confirming" \| "gender-confirming" \| "gender-affirming" \| "gender affirming" \| "gender reassignment" \| "transition-related" \| "sex reassignment" \| "gender change" \| "sex change" \| "transgender") AND ("surgery" \| "surgeries") AND "Canada" AND ("Quebec" \| "QB" \| "Q.B."\| ) | 187 |
| Ontario | ("gender confirmation" \| "gender confirming" \| "gender-affirming" \| "gender affirming" \| "gender reassignment" \| "transition-related" \| "sex reassignment" \| "gender change" \| "sex change" \| "transgender") AND ("surgery" \| "surgeries") AND Canada AND Ontario | 176 |
| Manitoba | ("gender confirmation" \| "gender confirming" \| "gender-confirming" \| "gender-affirming" \| "gender affirming" \| "gender reassignment" \| "transition-related" \| "sex reassignment" \| "gender change" \| "sex change" \| "transgender") AND ("surgery" \| "surgeries") AND "Canada" AND ("Manitoba") | 213 |
| Saskatchewan | ("gender confirmation" \| "gender confirming" \| "gender-confirming" \| "gender-affirming" \| "gender affirming" \| "gender reassignment" \| "transition-related" \| "sex reassignment" \| "gender change" \| "sex change" \| "transgender") AND ("surgery" \| "surgeries") AND "Canada" AND ("Saskatchewan" \| "Sask." ) | 170 |
| Alberta | ("gender confirmation" \| "gender confirming" \| "gender-confirming" \| "gender-affirming" \| "gender affirming" \| "gender reassignment" \| "transition-related" \| "sex reassignment" \| "gender change" \| "sex change" \| "transgender") AND ("surgery" \| "surgeries") AND "Canada" AND ("Alberta") | 173 |
| British Columbia | ("gender confirmation" \| "gender confirming" \| "gender-confirming" \| "gender-affirming" \| "gender affirming" \| "gender reassignment" \| "transition-related" \| "sex reassignment" \| "gender change" \| "sex change" \| "transgender") AND ("surgery" \| "surgeries") AND "Canada" AND ("British Columbia" \| "BC" \| "B.C.") | 162 |
| Yukon | ("gender confirmation" \| "gender confirming" \| "gender-confirming" \| "gender-affirming" \| "gender affirming" \| "gender reassignment" \| "transition-related" \| "sex reassignment" \| "gender change" \| "sex change" \| "transgender") AND ("surgery" \| "surgeries") AND "Canada" AND ("Yukon") | 208 |
| Northwest Territories | ("gender confirmation" \| "gender confirming" \| "gender-confirming" \| "gender-affirming" \| "gender affirming" \| "gender reassignment" \| "transition-related" \| "sex reassignment" \| "gender change" \| "sex change" \| "transgender") AND ("surgery" \| "surgeries") AND "Canada" AND ("Yukon") | 205 |
| Nunavut | ("gender confirmation" \| "gender confirming" \| "gender-confirming" \| "gender-affirming" \| "gender affirming" \| "gender reassignment" \| "transition-related" \| "sex reassignment" \| "gender change" \| "sex change" \| "transgender") AND ("surgery" \| "surgeries") AND "Canada" AND ("Nunavut") | 197 |

Work Cited

[1. Livoreil B, Glanville J, Haddaway NR, Bayliss H, Bethel A, de Lachapelle FF, et al. Systematic searching for environmental evidence using multiple tools and sources. Environ Evid. 2017 Aug 21;6(1):23.](https://www.zotero.org/google-docs/?cApkpP)

[2. Mahood Q, Van Eerd D, Irvin E. Searching for grey literature for systematic reviews: challenges and benefits. Res Synth Methods. 2014;5(3):221–34.](https://www.zotero.org/google-docs/?cApkpP)

[3. UK LMP. Covidence. 2021 [cited 2024 May 9]. How to write a search strategy for your systematic review. Available from: https://www.covidence.org/blog/how-to-write-a-search-strategy-for-your-systematic-review/](https://www.zotero.org/google-docs/?cApkpP)
